# Color call improvement in next generation sequencing using multi-class support vector machines

**DOI:** 10.1186/1471-2105-13-S18-A3

**Published:** 2012-12-14

**Authors:** Shruthi Viswanath, Chengyong Yang

**Affiliations:** 1 Department of Computer Science, University of Texas, Austin, Texas, USA; 2 Life Technologies Inc, CA, USA

## Background

There is considerable ongoing effort towards making DNA sequencing machines faster and more affordable today. Improving the accuracy of next-generation sequencers directly lowers sequencing costs by reducing the need for resequencing, making genome-based diagnostics and research more affordable [1]. In this paper, we show how the accuracy of next-generation sequencing machines is significantly improved using supervised learning, specifically, multi-class support vector machines. We demonstrate our methods on the SOLiD 5500/5500 XL platform.

Base-calling is the process of determining the order of nucleotides in the read sequence. In SOLiD, base-calling involves the process of color calling, since the SOLiD platform uses an encoding system where each adjacent pair of nucleotides is represented by one of four colored dyes [2]. Base-callers have been developed for other next-generation sequencing platforms, in particular Illumina and Roche 454 [1]. Most of them are based on explicit statistical models and some are based on support vector based supervised learning [3,4]. But ours is the first supervised learning method applied on a large scale directly to color space. Also, this is the first supervised learning method to be applied on a large-scale to SOLiD. Moreover, we show that our methods require less training data and hence our training times are much faster than previous methods.

## Materials and methods

Noise in sequencing is due to the imperfect nature of the chemical processes involved. Specifically, incomplete cleavage of bases from previous cycles results in residual signal, a problem known as phasing. Also, signal strength diminishes along the sequence due to depletion of chemicals. These errors accumulate over the sequence length, leading to lower accuracy at the end of a read sequence. We improve the sequencing accuracy by modeling these sources of error explicitly through support vector machines.

We represent the classification problem as one that takes as input, the raw color intensities of the current cycle (or sequence position) and presents as output, the color for that cycle. We use the raw dye intensities like [3], since, by doing so, we do not need to know each source of error explicitly, and the method will be more general and applicable to future releases, and different platforms. To address the phasing problem, we use not only the current cycle color intensities but also the previous cycle color intensities as input for the classifier. To account for depletion of chemicals, we train a separate classifier for each position in the read sequence. We use the SVMLight Multi-class package [5] with polynomial kernel and slack rescaling to test our methods.

## Results and conclusions

**Figure 1:**
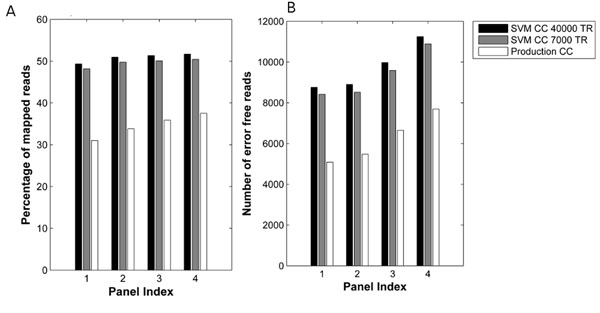
We show, for four panels of an E-coli run, the performance in terms of percentage of mapped reads (A) and number of error free reads (B), for an SVM Color Caller trained on 40000 reads (labeled SVM CC: 40000 reads) and one that is trained on 7000 reads per panel (labeled SVM CC: 7000 training reads). Both the SVM Color Callers are superior to the current SOLiD production color caller (labeled Production CC) and the SVM CC trained on 7000 reads is almost as good as the one trained on 40000 reads.

We tested our methods on the *Escherichia coli* genome dataset on the SOLiD 5500/5500XL platform. We noted that using the previous cycle intensities as additional inputs lowered the training error rate. Each sequencing run of E-coli had about 35 panels, with each of them consisting of about 100,000 reads. We observed that we were able to train on as little as 7000 reads of one single panel per lane, and apply the training for classifying all the rest of the reads in all other panels in that lane. In other words, the training is highly generalizable not only to other sample reads in the same panel, but also to other panels in the lane. We outperformed the current SOLiD color caller significantly in terms of percentage of reads that were correctly mapped to the genome, and number of error-free reads. Figure 1 shows the performance of the supervised color caller and current SOLiD color caller for four panels of a single lane. The method is fast, with training times of 1-2 min per lane. A future application would be to use the supervised learning to train on control reads and apply the training to sample reads. Incorporating this method would considerably improve next-generation sequencers.
